# Prebiotic attenuation of olanzapine-induced weight gain in rats: analysis of central and peripheral biomarkers and gut microbiota

**DOI:** 10.1038/s41398-018-0116-8

**Published:** 2018-03-15

**Authors:** Amy Chia-Ching Kao, Sonia Spitzer, Daniel C. Anthony, Belinda Lennox, Philip W. J. Burnet

**Affiliations:** 1 Department of Psychiatry, University of Oxford, Oxford, OX3 7JX UK; 2 Department of Pharmacology, University of Oxford, Oxford, OX1 3QT UK

## Abstract

Olanzapine is an effective antipsychotic drug but since it causes significant weight gain, it is not well tolerated by psychosis patients. The prebiotic, B-GOS^®^, attenuates metabolic dysfunction in obese subjects, and in rodents, alters central NMDA receptors and may affect serotonin receptors that are relevant in psychosis. We have determined whether B-GOS^®^ influenced olanzapine-associated weight gain and central NMDA and serotonin receptors. Circulating acetate, IL-1β, IL-8 and TNFα, liver acetyl-CoA carboxylase (ACC), white adipose tissue (WAT) acetate receptor GPR43, and specific faecal bacteria genera were also measured to provide mechanistic information. Adult female Sprague-Dawley rats were administered a B-GOS^®^ (0.5 g/kg/day) solution or water for 21 days, and received a single, daily, intraperitoneal injection of olanzapine or saline on days 8–21. The intake of B-GOS^®^ significantly attenuated olanzapine-induced weight gain without altering frontal cortex 5-HT2AR blockade. Cortical GluN1 levels were elevated by olanzapine in the presence of B-GOS^®^. Plasma acetate concentrations increased following B-GOS^®^ or olanzapine administration alone, but reduced when prebiotic and drug were administered in combination. This pattern was paralleled by hepatic ACC mRNA expression. The abundance of WAT GPR43 mRNA was reduced by olanzapine, only in the absence of B-GOS^®^. Co-administration of B-GOS^®^ and olanzapine also elevated plasma TNFα, which is reported to influence lipid metabolism. Finally, B-GOS^®^ elevated faecal *Bifidobacterium *spp. and reduced some bacteria in the Firmicutes phylum, whilst olanzapine treatment either alone or with B-GOS^®^, was without effect. These data suggest that inclusion of B-GOS^®^ as an adjunct to olanzapine treatment in schizophrenia may prevent weight gain and have benefits on cognitive function in psychosis. The role of acetate in these effects requires further investigation.

## Introduction

Olanzapine is a second-generation antipsychotic medication that is effective at treating the positive symptoms of psychosis and preventing future relapse^1^. Current clinical guidelines recommend treatment with antipsychotics for at least a couple of years after symptomatic improvement. Weight gain is a prominent side effect of second-generation antipsychotics, particularly clozapine and olanzapine^2,3^. In the treatment of schizophrenia, olanzapine robustly confers more than 7% increase in body weight in patients within the first year of treatment^4–6^. This weight gain not only affects the tolerability of the medication, but also presents as a significant risk factor for metabolic syndrome and cardiovascular disease in patients, and contributes to the significant morbidity and mortality associated with psychotic illnesses. Cognitive impairment is consistently described in patients with schizophrenia, even at first episode of illness, which is not improved and may even be potentially exacerbated by treatment with olanzapine^7^. Therefore, an adjunctive intervention that reduces the antipsychotic-mediated weight gain without compromising the antipsychotic effect of the medication, whilst potentially improving the cognitive deficits seen in schizophrenia, would be therapeutically beneficial.

The variations in binding affinities of second-generation antipsychotic medication to dopaminergic and serotoninergic receptors represent a key pharmacological parameter that differentiates them from the first-generation antipsychotics, and is often attributed to their clinical effects^8^. In rodents, prolonged administration of olanzapine led to decreased binding densities of brain serotonin 2A receptors (5-HT2AR) and 5-HT1AR^9^, and to a lesser extent 5-HT2CR^10^. This effect is considered important for the therapeutic actions of olanzapine on both positive and negative symptoms of schizophrenia, although the mechanisms underlying cognitive impairments, and how they are affected by olanzapine is not well understood. One pervasive hypothesis of schizophrenia is the disrupted function of the glutamate N-methyl-D-aspartate receptor (NMDAR), an ionotropic receptor that is composed of two obligatory GluN1 subunits and two regulatory GluN2A and/or GluN2B subunits, and in some cells, GluN3A.

Using NMDAR antagonists, studies have demonstrated that optimal NMDAR function is crucial for healthy neurodevelopment, learning and memory in rodents^11–14^, and partial blockade induces a range of psychiatric and neurocognitive symptoms in healthy individuals^15^. Consideration of NMDAR activity and function is important for schizophrenia pharmacotherapy as reductions in NMDAR levels in rodent brains were reported following olanzapine administration, possibly contributing and exacerbating cognitive deficits^16–18^. Notably, these studies were performed in male rats which are not susceptible to olanzapine-mediated weight gain^19–21^, and in some cases were not performed at clinically relevant doses of the drug (i.e., less than 7.5 mg/kg/day)^22,23^.

Recent studies have shown that augmenting gut bacteria influences central glutamate neurotransmission, host body weight, metabolism, and systemic inflammation^24,25^. Faecal microbiota analysis in female rats following 21 days of olanzapine treatment revealed significant shifts in phyla composition that accompanied the significant weight gain^19,26^. Such shifts include the increased abundance of the major phyla Firmicutes, reductions in Bacteriodetes, as well as reduced diversity of Actinobacteria and Proteobacteria. Given that previous studies in germ-free mice suggest the relative composition of the gut microbiome may underlie diet-induced obesity^27,28^, targeting the microbiome may present as a potential strategy to attenuate or ameliorate drug-induced weight gain. In studies examining risperidone-induced weight gain, suppressed energy expenditure has been posited as a contributor to overall weight gain in both wildtype female mice^29^ and children^30^. Alternatively, weight gain may occur through the immune system as circulating cytokines, in particular TNFα, has recently been shown to influence weight gain by regulating lipid metabolism^31^ and appetite suppression^32^. Furthermore, the administration of probiotics (live cultures of beneficial bacteria) and prebiotics (dietary fibres that grow indigenous good bacteria) in obese and overweight adults resulted in corrections of key metabolic parameters (e.g., body weight, body mass index) and serum markers (e.g., cytokines, acetate)^33,34^.

The microbial fermentation of prebiotics, such as Bimuno^TM^ galacto-oligosaccharides (B-GOS^®^), produces the short-chain fatty acids (SCFA) including acetate, butyrate, and propionate^35,36^, which themselves influence host physiology^37^. Importantly, the transport of acetate into glial cells is modulated by signal transmissions dependent on the NMDAR^38^, and acetate supplementation itself elevates brain NMDAR subunit levels^39^ and rescues NMDAR hypofunction in rodents^40^. Circulating acetate is also an appetite suppressant^41^, and increased hepatic levels of its metabolizing enzyme, acetyl CoA carboxylase (ACC) has been associated with reduced weight gain in olanzapine-injected rats, following antibiotic administration^26^. Furthermore, reduced expression of the acetate binding receptor GPR43 in adipose tissue has been associated with high-fat diet-mediated weight gain in mice, and supplementation of SCFA, including acetate alone, restores GPR43 abundance to control levels^42^. Together, all the above observations suggest that prebiotic fermentation by gut microbiota may offer a safe and promising approach to improve the metabolic health of schizophrenia patients treated with atypical antipsychotics, and may have additional effects on central NMDAR function.

The aim of this study was therefore: (1) to test if the prebiotic B-GOS^®^ attenuated olanzapine-induced weight gain in female rats without compromising the central pharmacological action of the drug. (2) To evaluate whether B-GOS^®^ influenced the actions of olanzapine on cortical and hippocampal NMDAR subunit proteins and transcripts. (3) To explore the influence of B-GOS^®^ and olanzapine on the concentration of plasma inflammatory markers and acetate, and the abundance of hepatic ACC, white adipose tissue (WAT) GRP43, and specific faecal gut microbial genera.

## Materials and methods

### Materials

Clasado BioSciences Ltd., UK, supplied commercially available, purified prebiotic Bimuno™ galacto-oligosaccharides (B-GOS^®^) powder. Custom DNA oligomers were purchased from Eurofins Genomics, UK. Olanzapine was purchased from Oxford Pharmacy Stores UK, and dissolved at 10 mg/ml in sterile saline solution (0.9% NaCl) adjusted to pH 6.5 with concentrated hydrochloric acid. Rat IL-1β and TNFα Quantikine Enzyme-Linked Immunosorbent Assays (ELISA) were purchased from Bio-techne, UK; and IL-8 Sandwich ELISA kit from 2BScientific, UK. Colorimetric acetate assay kit was purchased from Abcam, UK.

### Animals

Female adult Sprague-Dawley rats (*n* = 24), 220–250 g (6–8wks), were housed three per cage with rodent chow and water *ad libitum* on a 12 h light-dark cycle (21 ± 1 °C, humidity 50 ± 5%). Female rats only were used in this study because, olanzapine-induced weight gain is not observed in male rats^19,20^. All procedures were performed in accordance with UK Home Office Animals (Scientific Procedures) Act (1986) and associated Home Office guidelines. The local Animal Welfare and Ethical Review Body (AWERB) at the University of Oxford approved the procedures specific to this study.

### Drug and prebiotic administration

Rodents were randomly assigned to one of four different treatment groups: saline/water, B-GOS^®^/saline, water/olanzapine, B-GOS^®^/olanzapine. Depending on group, rats were provided with water or water supplemented with B-GOS^®^ (0.5 g/kg/day) for 1 week. This was followed by a 2-week, daily intraperitoneal injection of olanzapine (10 mg/kg) or saline, during which water or B-GOS^®^ administration continued. This dose of olanzapine was chosen because of its relevance to clinical use. That is, occupancy of target central receptors with olanzapine in rodents occurs above 7.5 mg/kg following repeated administrations, with full saturation achieved at 10 mg/kg^22,23^. This is because, the half-life of olanzapine in rodents is 4–6 times greater than in humans. The repeated administration of lower doses (2–4 mg/kg) of the drug to rats are sufficient to model antipsychotic induced metabolic dysfunction^19,26^, but may limit studies of olanzapine’s central actions. Intraperitoneal injection of the drug is a standard route of delivery to rodents to ensure all animals receive the same dose, and is consistent with other olanzapine studies^19,26^. Quantity of fluid intake and weight gain were monitored daily. Weights were normalised to the day of olanzapine administration in order to model the olanzapine effect and were expressed as percentage weight gain (i.e., [weight – weight at the start of olanzapine administration / weight at the start of olanzapine administration] x 100).

### Plasma and tissue collection

Twenty four hours after the final injection, animals were humanely culled by cervical dislocation and decapitated to obtain trunk blood, which was collected in ethylenediaminetetraacetic acid (EDTA) coated 2 ml Eppendorf tubes. All blood samples were centrifuged at maximum speed on a bench-top centrifuge for 5 min. The top plasma phase was pipetted into a fresh Eppendorf tube and frozen on dry ice. Whole brains were removed and bisected into left and right hemispheres on ice. One-half was flash frozen in isopentane on dry ice for cryosectioning, while the other was dissected to obtain frontal cortex and hippocampal tissues for immunoblotting. All plasma and tissue samples were stored in −80 °C until analysed.

### Immunoblotting

Immunoblotting was carried out as previously described^43^. Briefly, brain tissues were homogenised in RIPA buffer (Sigma-Aldrich, UK) containing 0.1% (v/v) protease inhibitor cocktail. Protein concentrations of tissue homogenates were determined using Bradford’s protein assay (Bio-rad laboratories, CA, USA). Total protein (5–50 μg) were loaded into a precast 4–20% Mini-Protean TGX Gel (Bio-Rad) and separated with SDS-PAGE in 1 × Running Buffer (25 mM Tris base, 250 mM Glycine, 0.1% SDS, diH_2_O). Proteins were then transferred to a polyvinylidene difluoride (PVDF) membrane overnight using electroblotting in 1 × Transfer Buffer (25 mM Tris base, 192 mM Glycine, 20% Methanol, diH_2_O). The membrane blots were reactivated in methanol and incubated for 1 h at room temperature in blocking buffer (0.1% PBS-Tween20 with 5% skim milk). Primary and secondary antibodies were prepared in 0.1% PBS-T with 2% milk and added to blots sequentially for 1 h and 40 min respectively, separated by three 20 min washes in Tris-buffered saline with PBS-T. Immunoreactivity was visualized with enhanced chemiluminescence (Amersham ECL Prime) and exposure to Hyperfilm (Amersham) at various time points. Densitometric analysis was performed using AlphaImager 3400 to quantify relative protein expression using film exposures below the saturation point. β-Actin was used as an internal control to normalize the optical density values in each sample.

Antibodies used in western blots included anti-GluN1 (1:5000, Millipore), anti-GluN2A (1:5000, Millipore), anti-GluN2B (1:5000, Millipore), anti-5-HT2AR (1:1000, Abcam), anti-5-HT1AR (1:1000, Abcam) and anti-β-Actin (1:1000000, Sigma). Horseradish peroxidase-conjugated goat anti-rabbit (1:10000, Bio-Rad) and goat anti-mouse (1:5000, Biorad) were used as secondary antibodies.

### In situ hybridization histochemistry (ISHH)

Fourteen micron sagittal sections (lateral 2.90 mm, bregma 0.48 mm, interaural 9.48 mm) of frozen rat brain hemispheres were collected on glass slides (3 sections/slide) and subjected to pre-hybridization treatment using an established ISHH protocol as previously described^44^. Oligodeoxyribonucleotide cDNA probes complementary to: GluN1 (bases 746–780, NM008169.1), GluN2A (bases 1642–1676, NM008170.2) or GluN2B (bases 1758–1792, NM010350.2) were 3′-end labelled with [^35^S]-dATP using terminal deoxynucleotidyl transferase (Promega, UK). Probes were then diluted in hybridization buffer, pipetted onto the tissue sections (1 × 10^6^ cpm/section), cover-slipped and then incubated for over 16 h at 34 °C in lidded Perspex trays lined with filter paper soaked with 4× SSC/50% formamide. Post-hybridization washes included 2× SSC rinse at room temperature to remove cover-slips, 3× in 0.5× SSC for 20 min at 55 °C, and 2× in 0.5× SSC for 30 min at room temperature. Slides were rinsed in ddH_2_O, dried and exposed to X-ray film (Kodak, Biomax MS) for 7–28 days with ^14^C-microscales. Average grey densities over the frontal cortex, and dentate gyrus, CA1 and CA3 subfields of the hippocampus in the three sections from each group were measured using computer-assisted image analysis. Densities were calibrated to ^35^S nCi/g tissue equivalents using commercial ^14^C-microscales.

### Plasma analysis

Levels of circulating inflammatory markers and acetate were measured using commercially available assay kits (see above). The concentrations of IL-1β and IL-8 were of particular interest since they have been shown to be affected by olanzapine administration in female rats^19^. All samples were assayed in duplicate and protocols were carried out according to manufacturer’s instructions.

### Extraction of liver and WAT RNA and quantitative PCR (QPCR)

Total RNA was extracted from the remaining fragment of cortical tissue using Tri-Reagent according to manufacturer’s instructions, and reverse-transcribed to cDNA, using a commercial kit (Life Technologies, UK). The SYBR Green methodology (Power SYBER, Life Technologies, UK) was applied to amplify mRNA encoding ACC and GPR43 in liver and WAT cDNA preparations, respectively, using previously published primer sets. The QPCR was performed on a 7900HT Fast Real-Time PCR System (Applied Biosystems, USA). All QPCR data are presented as fold change (relative to β-2-microglobulin, B2M) which were calculated using the 2^-ΔΔCt^ relative quantitation method^45^.

### Gut microbiota analysis

The abundance of 16S DNA encoding specific microbial genera and total bacteria in faecal pellets were analysed using QPCR and previously published primers^46^. Bacterial DNA was extracted using QIAamp DNA Stool Mini Kit (Qiagen, Hilden, Germany) after mechanical disruption. The concentrations of isolated DNA were quantified spectrophotometrically by measuring absorbance at 260 nm. The SYBR Green methodology (Power SYBER, Life Technologies, UK) was used to amplify 20 ng DNA on a 7900HT Fast Real-Time PCR System (Applied Biosystems, US): duplicate samples were held at 95 °C for 10 min and subjected to 40 cycles of denaturation (95 °C for 15 s) and annealing/extension (60 °C for 60 s). These QPCR data were also calculated using the 2^−ΔΔCt^ relative quantitation method^45^ and reported as fold-changes relative to the saline/water group.

### Statistical analyses

Statistical analyses were performed using SPSS, version 22. Normal distribution of data was confirmed using Shapiro-Wilk test. All data are expressed as mean + standard error of the mean (SEM) unless otherwise stated. Body weight data were analysed with two-way ANOVA (repeated measures), comparing time (day), ‘diet’ (water, B-GOS^®^) and ‘treatment’ (saline, olanzapine), followed by post hoc Bonferroni correction when significance was reached. Western blot optical density ratios, mRNA abundance and plasma changes were analysed using 2-way ANOVA with Bonferroni correction. Microbial data were analysed with non-parametric Kruskall-Wallis test, and Mann-Whitney U post hoc comparisons. The level of significance was set at 0.05 and *p*-values and *F*-statistics are reported as exact values.

## Results

### Effect of B-GOS^®^ on olanzapine-induced weight gain

**Fig. 1:**
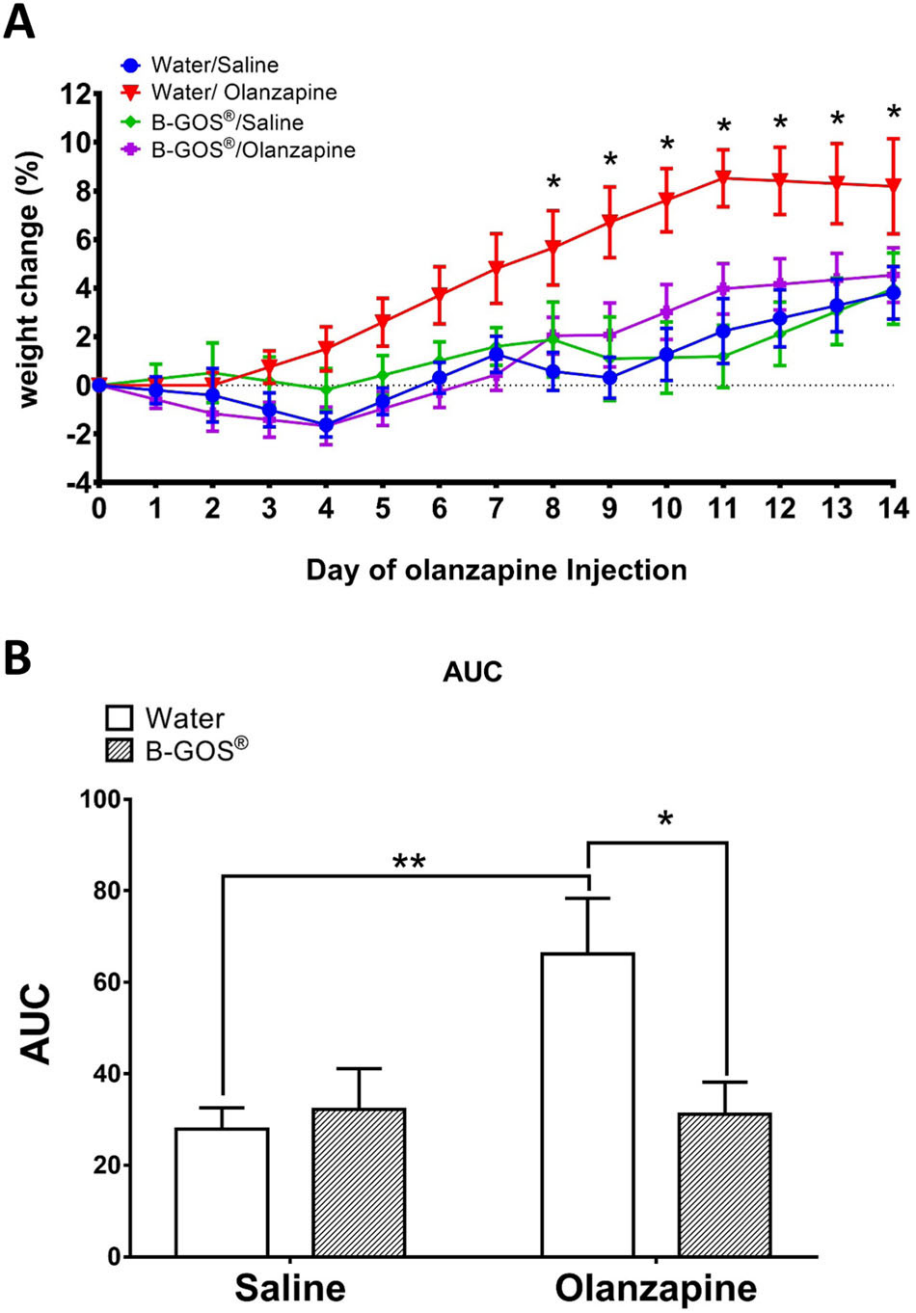
Effect of B-GOS^®^ (0.5 g/kg/day) ingestion and olanzapine (10 mg/kg/day) administration on weight gain in adult female rats. Animals ingested B-GOS^® ^7 days prior to, and throughout the 2-week administration of olanzapine. **a** Percentage weight gain was calculated from the day before olanzapine administration. **b** Area under the curve analysis. Results are expressed as mean ± SEM for each group (*n* = 6); *p < 0.05, compared to the saline/olanzapine group

A 2-week intraperitoneal olanzapine administration to female rats increased body weight compared to saline injected animals, and this was attenuated by B-GOS^®^ (Fig. 1). A significant time × treatment interaction (*F*_13, 260_ = 7.958; *p* < 0.001) was observed, and post hoc analysis revealed that this was driven by weight gain in water/olanzapine rats on days 8–14 (*p* < 0.05) (Fig. 1a). There was also a significant diet × treatment interaction (F_1,20 = _5.102; *p* = 0.035), where overall weight gain in B-GOS^®^/olanzapine rats was less than water/olanzapine animals (F_1,20 = _7.467; *p* = 0.013). A time × diet × treatment effect was not observed (*F*_13,260_ = 1.124; *p* > 0.05) which indicated that B-GOS did not influence overall weight gain during the two week olanzapine and saline injections.

The analysis of area under the curve (Fig. 1b) illustrated the diet × treatment interaction (*F*_1,20_ = 4.629; *p* = 0.044). Water/olanzapine animals experienced significantly greater weight gain than water/saline controls (*p* = 0.005); this change was attenuated by B-GOS^® ^(water/olanzapine vs B-GOS^®^/olanzapine, *p* = 0.01).

### Effect of olanzapine and B-GOS^®^ on cortical 5-HT receptors and GluN subunits

**Fig. 2:**
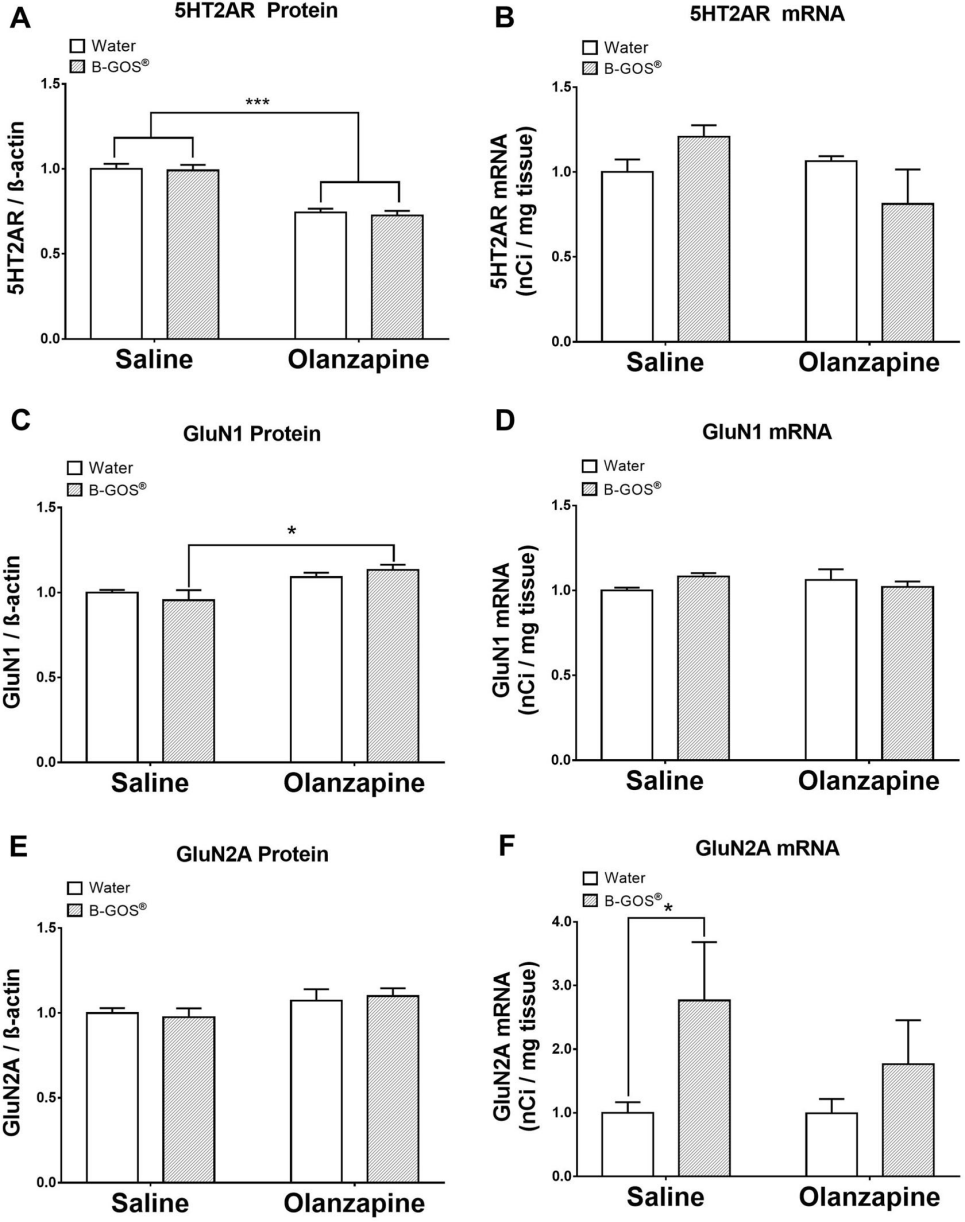
Effect of B-GOS^®^ and olanzapine administration in adult female rats on the cortical protein and gene expression of (a, b) 5-HT2AR, (c, d) GluN1 subunit, and (e, f) GluN2A subunit, respectively. Animals ingested B-GOS^®^ 7 days prior to, and throughout, the 2-week administration of olanzapine. Results are expressed as mean ± SEM for each group (*n* = 6). *p < 0.05

There was a significant effect of treatment (*F*_1,17_ = 64.582; *p* < 0.001) on cortical 5-HT2AR protein levels where a ~ 30% decrease was observed in the olanzapine-treated group (independent of B-GOS^®^ supplementation) (Fig. 2a). No significant effects were observed in 5-HT2AR mRNA abundance, though a diet × treatment trend (*F*_1,17_ = 4.403; *p* = 0.051) was noted. There was no significant effect of olanzapine or B-GOS^®^ on cortical 5-HT1AR protein (water/saline 0.79 ± 0.265, B-GOS^®^/saline 0.83 ± 0.204, water/olanzapine 0.82 ± 0.278, B-GOS^®^/olanzapine 0.91 ± 0.106 [5-HT1AR/β-actin]; *p* > 0.05); or mRNA (water/saline 169.53 ± 68.887, B-GOS^®^/saline 366.2 ± 223.651, water/olanzapine 200.12 ± 93.634, B-GOS^®^/olanzapine 249.76 ± 158.302 [nCi/mg tissue]; *p* > 0.05).

An overall effect of treatment (*F*_1,20_ = 8.683; *p* = 0.01) on cortical levels of GluN1 was detected (Fig. 2c). Post hoc analysis revealed that olanzapine increased GluN1 levels in B-GOS^®^-fed rats only (olanzapine/B-GOS^®^ vs olanzapine/water: +13%; *F*_1,20_ = 11.723; *p* = 0.028), whilst mRNA levels remained comparable between all groups (Fig. 2d). A similar effect was observed for GluN2B protein, though statistical significance was not reached (*F*_1,20_ = 3.622; *p* = 0.072; data not shown). However, cortical GluN2A mRNA expression was significantly affected by diet (*F*_1,19_ = 5.638; *p* = 0.031), which was mainly driven by greater transcript levels in B-GOS^®^/saline rats compared to the water/saline group (*F*_1,19_ = 4.838; *p* = 0.047) (Fig. 2f).

In the hippocampal sub-regions (Table 1), there was a significant effect of treatment on GluN1 mRNA abundance in the CA3 (*F*_1,19_ = 5.464; *p* = 0.034), where olanzapine significantly increased (+17%) levels in the absence of B-GOS^®^ (*F*_1,19_ = 4.988; *p* = 0.040). No significant main effects of treatment or diet were observed on GluN2A, GluN2B or 5-HT1AR protein and mRNA levels in the hippocampus.

**Table 1:** Effect of prebiotic B-GOS^®^ (0.5 g/kg/day) ingestion and olanzapine (10 mg/kg/day) administration in adult female Sprague-Dawley rats on the hippocampal protein levels and gene expression of central receptor and subunits

Receptor			Mean ± SEM [protein / β-actin] & [mRNA (nCi/mg tissue)]			
			Saline		Olanzapine	
	Region	Parameter	Water	B-GOS^®^	Water	B-GOS^®^
5-HT1A	Hippocampus	Protein	1.28 ± 0.10	1.28 ± 0.06	1.14 ± 0.08	1.14 ± 0.05
	CA1	mRNA	518.83 ± 43.66	741.59 ± 104.21	522.14 ± 43.62	618.16 ± 87.84
	CA3	mRNA	362.51 ± 40.81	545.7 ± 111.44	541.09 ± 33.57	457.38 ± 109.72
	DG	mRNA	865.73 ± 55.97	1047.54 ± 137.64	981.64 ± 36.24	1067.72 ± 143.93
GluN1	Hippocampus	Protein	0.52 ± 0.02	3.05 ± 1.37	0.53 ± 0.01	0.52 ± 0.02
	CA1	mRNA	537.2 ± 37.39	524.83 ± 18.20	525.3 ± 16.45	545.8 ± 19.59
	CA3	mRNA	428.01 ± 9.50	402.13 ± 23.08	499.55 ± 35.18*	450.34 ± 16.32
	DG	mRNA	421.71 ± 20.48	413.49 ± 22.64	457.07 ± 22.36	444.53 ± 13.37
GluN2A	Hippocampus	Protein	0.87 ± 0.02	0.91 ± 0.03	0.89 ± 0.03	0.88 ± 0.03
	CA1	mRNA	1889.73 ± 196.92	2318.98 ± 223.33	1877.17 ± 138.43	2116.8 ± 204.93
	CA3	mRNA	962.7 ± 156.67	1087.6 ± 220.15	937.25 ± 116.42	1011.88 ± 198.56
	DG	mRNA	1353.34 ± 221.58	1584.36 ± 223.71	1276.06 ± 116.88	1394.5 ± 202.46
GluN2B	Hippocampus	Protein	0.89 ± 0.02	0.93 ± 0.02	0.92 ± 0.02	0.89 ± 0.02
	CA1	mRNA	1823.92 ± 193.99	1647.21 ± 174.81	2035.27 ± 130.60	1815.84 ± 167.53
	CA3	mRNA	1151.33 ± 143.25	960.15 ± 175.99	1225.19 ± 112.32	1072.1 ± 118.32
	DG	mRNA	1597.51 ± 163.72	1489.14 ± 186.00	1828.89 ± 147.58	1543.75 ± 135.73

*p < 0.05 compared to water/saline controls

### Effect of olanzapine and B-GOS^®^ on peripheral markers

A significant diet × treatment interaction was observed for the circulating levels of acetate (*F*_1,20_ = 15.341; *p* = 0.001), liver ACC mRNA (F_1,22 = _5.384; *p* = 0.032) and WAT GPR43 mRNA (*F*_1,22 = _7.592; *p* = 0.014) (Table 2). A post hoc analysis revealed that B-GOS^®^ significantly increased plasma acetate concentrations by approximately two-fold (water/saline vs B-GOS^®^/saline, *p* = 0.005). Similarly, the administration of olanzapine alone significantly increased circulating acetate levels by 1.78-fold (water/saline vs water/olanzapine, *p* = 0.037). The combined administration of olanzapine and B-GOS^®^, however, decreased acetate to control levels (water/olanzapine vs B-GOS^®^/olanzapine, *p* = 0.029). Subsequent post hoc analysis of ACC mRNA did not detect a significant difference between groups (water/saline vs B-GOS^®^/saline, *p* = 0.089; water/olanzapine vs B-GOS^®^/olanzapine, *p* = 0.117), whereas WAT GPR43 mRNA abundance in the B-GOS^®^/olanzapine group was significantly greater than in the water/olanzapine rats (*P* = 0.026). Although B-GOS^®^ alone reduced GPR43 mRNA to a magnitude similar to that seen in water/olanzapine animals (~ −70%), significance was not reached (water/saline vs water B-GOS^®^, *p* = 0.153).

**Table 2:** Effect of B-GOS (0.5 g/kg/day) ingestion and olanzapine (10 mg/kg/day) administration in adult female Sprague-Dawley rats on the levels of peripheral markers

Plasma/tissue marker	Mean ± SEM			
	Saline		Olanzapine	
	Water	B-GOS	Water	B-GOS
Acetate (μmol/L)^*^	111 ± 23	234 ± 37^**^	197 ± 19^**^	106 ± 28^***^
IL-1β (pg/ml)	1163.16 ± 17.68	1188.37 ± 25.93	1217.52 ± 48.29	1174.13 ± 33.89
IL-8 (ng/ml)	261.73 ± 8.31	256.28 ± 9.66	261.58 ± 9.66	277.47 ± 11.55
TNFα (pg/ml)	8.52 ± 0.22	8.23 ± 0.26	8.98 ± 0.47^†^	9.22 ± 0.28^†^
Liver ACC mRNA (fold-change)^*^	1.00 ± 0.41	2.31 ± 0.95	2.21 ± 0.98	1.18 ± 0.76
WAT GPR43 mRNA (fold-change)^*^	1.00 ± 0.02	0.35 ± 0.08	0.42 ± 0.16	1.23 ± 0.42^**^

^*^p < 0.05, diet × treatment interaction; **p < 0.05 compared to water/saline; ***p < 0.05 compared to water/olanzapine; ^†^*p* < 0.05 overall effect of treatment

The measurement of some circulating inflammatory markers (Table 2), revealed a significant effect of treatment for the concentration of TNFα (*F*_1,19_ = 4.835, *p* = 0.037), and post hoc analysis revealed that this was driven by olanzapine administration increasing levels of circulating TNFα in the presence of B-GOS^®^ (B-GOS^®^/saline vs B-GOS^®^/olanzapine, *p* = 0.049).

### Faecal microbiota characterization

**Fig. 3:**
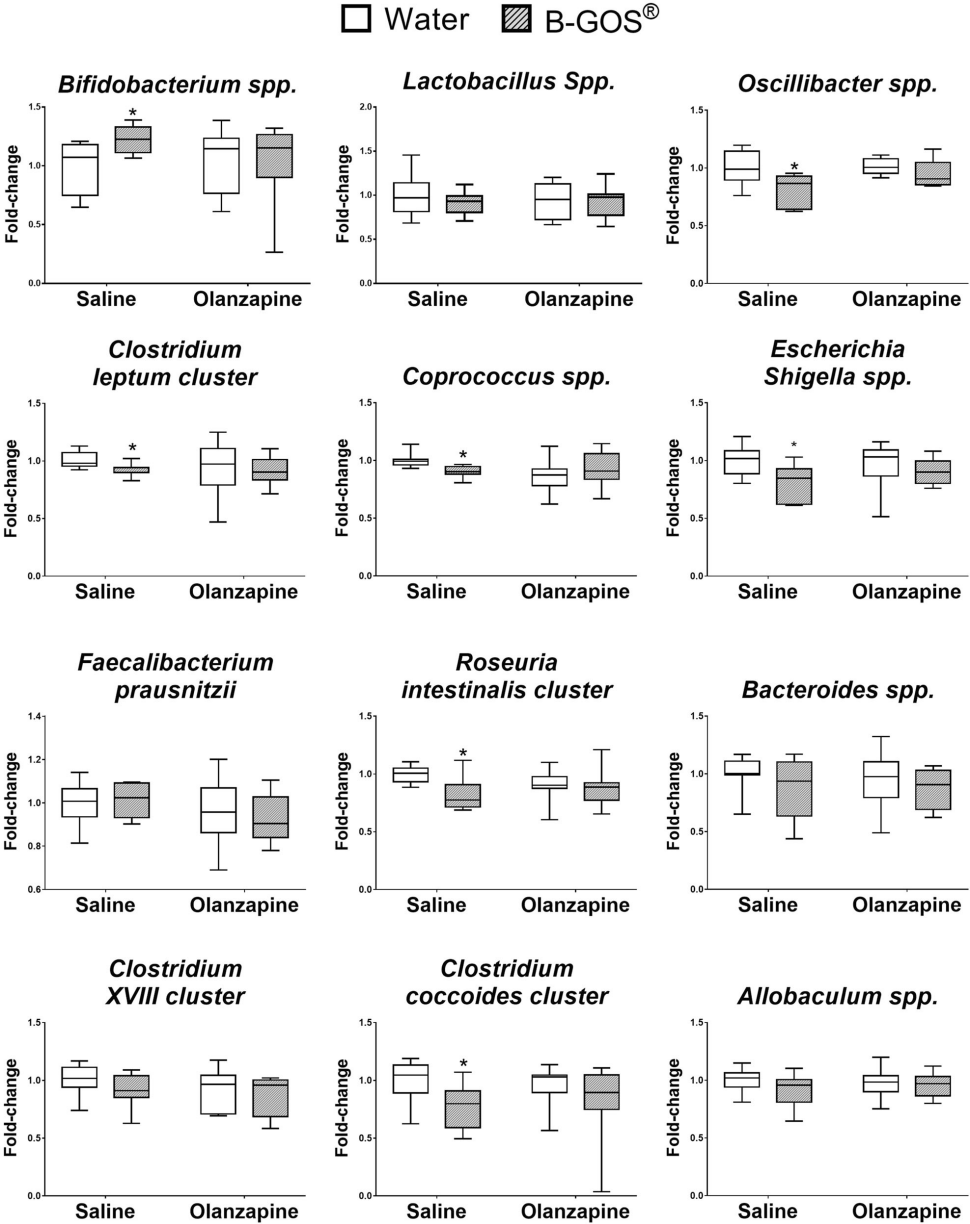
Effect of B-GOS^®^ and olanzapine administration on the faecal bacterial composition in female rats. Animals ingested B-GOS^®^ 7 days prior to, and throughout, the 2-week administration of olanzapine. Results are expressed as fold-change relative to the saline/water group, and presented with medians [first quartile to third quartile]. *p < 0.05, compared to the saline/water group

The intake of B-GOS^®^ increased *Bifidobacteria *spp. compared to water/saline animals (*p* = 0.026), corroborating previously published results in humans and rodents^47,48^. This increase was not observed in olanzapine-injected animals (water/olanzapine vs B-GOS^®^/olanzapine, *p* > 0.05). Other microbial genera including *Escherichia/Shigella *spp.,* Coprococcus* spp.,* Oscillibacter* spp.,* C. Coccoides* spp.,* Roseuria Intestinalis Cluster*, and *clostridium XVIII cluster* significantly decreased following the ingestion of B-GOS^®^ alone (Fig. 3). The administration of olanzapine did not affect faecal microbiota composition.

## Discussion

We have examined the effects of the prebiotic, B-GOS^®^, on olanzapine-induced weight gain, central 5-HTRs and NMDAR subunit levels in female rats. Our key finding was that B-GOS^®^ feeding attenuated olanzapine-induced weight gain without influencing the reduction of central 5-HT2AR levels. This study also demonstrated that olanzapine administration did not decrease NMDAR subunit gene expression, as previously reported in male rats^43^, but increased levels of GluN1 in the frontal cortex, in the presence of B-GOS^®^, and GluN1 mRNA expression in the CA3 region of the hippocampus. An analysis of rat plasma revealed that circulating acetate was increased by both B-GOS^®^ and olanzapine alone, but was reduced to control levels upon their co-administration. Significant B-GOS^®^ × olanzapine interactions for liver ACC and WAT GPR43 gene expression, confirmed the involvement of acetate metabolism in prebiotic and antipsychotic actions. Finally, olanzapine alone or with B-GOS^®^ did not affect the abundance of some specific genera of enteric bacteria, whilst B-GOS^®^ alone had some notable effects. Taken together, these data suggest that B-GOS^®^ may reduce weight gain when used adjunctively with second-generation antipsychotic drugs, without affecting their central molecular actions.

The B-GOS^®^ mediated reduction in olanzapine-induced weight gain is a novel finding. One previous study showed an attenuation of olanzapine-induced weight gain with antibiotics in female rats^26^, which may be at odds with our current findings. However, if we hypothesise that specific bacteria are involved in olanzapine-induced weight gain in the host, then both prebiotic and antibiotic intake could hinder the actions of undesirable microbes; the former by allowing other species to outgrow and restrict the proliferation of harmful bacteria, and the latter by directly eliminating them. The long-term use of antibiotics to curb medication-induced metabolic dysfunction is not a viable translational option as patients are likely to develop antibiotic resistance, a caveat noted by the authors of the study^26^. Therefore, the adjunctive use of B-GOS^®^ in the treatment of schizophrenia presents as a safe and natural strategy to attenuate metabolic side effects, and is unlikely to confound desired therapeutic actions. The antagonism of brain 5-HT2AR by olanzapine is an important pharmacological property that may underlie its psychotropic actions; in the current study this blockade was seen as a decrease in cortical 5-HT2AR levels (Fig. 2a). The intake of B-GOS^®^ did not interfere with this effect, and therefore shows that central actions of the prebiotic are independent of the serotonin system (see also 5-HT1AR data in Results section).

The administration of the satiety hormone GLP-1 to olanzapine-injected female rats has been shown to reduce metabolic dysfunction and weight gain^49,50^. The attenuation of weight gain by GLP-1R activation may be mediated, in part, by the elevation of brain 5-HT2AR as shown in one investigation^51^. This action is in contrast to the pharmacological effect of olanzapine, suggesting the co-administration of GLP-1 agonists, or its analogues, with olanzapine may negate the antipsychotic action of the drug. A more recent study, however, suggests that GLP-1 may modulate feeding behaviour via the activation of cortical NMDARs^52^. While this is an advantageous property given that NMDAR hypofunction has been postulated in the pathophysiology of schizophrenia^53^, the potential for GLP-1 to confound the key serotonergic pharmacological actions of olanzapine remains an issue. In the current study, not only have we shown that B-GOS^®^ attenuates weight gain without affecting olanzapine/5-HT2AR interactions, but also that it may have beneficial effects on NMDAR mediated brain functions, which we have consistently reported in male rodents^43,48^.

Earlier work has demonstrated that a 2-week olanzapine administration (2 mg/kg/day b.i.d) in rats did not alter cortical NMDAR receptor subunit levels^54^. We have corroborated this finding at a clinically relevant dose of olanzapine (10 mg/kg/day) in female rats. However, olanzapine did increase cortical GluN1 protein in B-GOS^®^ fed rats, and elevated GluN1 mRNA in the CA3 hippocampal subfield of the water/ olanzapine animals. One study reported that a 4-week administration of olanzapine at 10 mg/kg/day reduced GluN1 in the hippocampus but increased GluN2A^18^. The physiological relevance of olanzapine-induced elevation of GluN subunits in the hippocampus seen in both the current study and the chronic experiments are therefore, worthy of further examination.

The present study also explored a potential mediator of the central NMDAR enriching and weight suppressing effects of B-GOS^®^. We have recently demonstrated that the concentration of acetate, the SCFA predominantly produced by microbial fermentation of B-GOS^®^^55^, increases in the plasma of male rats following the ingestion of this prebiotic^39^. Furthermore, acetate supplementation itself is not only associated with increased levels of NMDAR subunits^39^, but has also been shown to rescue the behavioural effects of NMDAR blockade in rats^40^. We now show that B-GOS^®^ intake also increases plasma acetate in female rats (Table 2), though GluN2A (Fig. 2f), rather than GluN1^43^ or GluN2B^39^, were elevated in the cortex. Since acetate acts as an appetite suppressant either directly^41^ or via stimulation of anorectic hormones PYY and GLP-1^56^, we examined whether the increase in acetate was associated with B-GOS^®^ mediated reduction of olanzapine-induced weight gain. Contrary to our hypothesis, our results showed that a reduction of acetate appeared to be linked to the B-GOS^®^-mediated attenuation of olanzapine-induced weight gain, and that levels of this SCFA increased after olanzapine injections. These data imply that elevated acetate levels may contribute to olanzapine-mediated weight gain.

Feeding rats with a high-fat diet also led to increased plasma acetate levels, and this was strongly associated with metabolic dysfunction and weight gain^57^. This is consistent with our observation that olanzapine-induced weight gain was associated with an elevation in circulating acetate levels, and the normalisation of olanzapine rat body weight was accompanied by the reduction of plasma acetate. However, in the current study B-GOS^®^ also elevated plasma acetate levels but did not increase body weight. This may suggest that acetate does not mediate weight gain, and perhaps elevated acetate in olanzapine-treated rats was a consequence of altered metabolism wherein the satiety-inducing action of this SCFA was compromised^41^. A precedent for this is the elevation of the anorectic hormone leptin following olanzapine administration in rodents and patients^58^. In these cases, increased leptin may have been a homeostatic response to the medication-induced metabolic disturbance that fails to initiate its appetite supressing properties. Another complication in interpreting our findings are data showing that a high-fat diet decreases acetate, and that wheat fibre supplementation restores it to normal concentrations^59^. This is in keeping with our observation that B-GOS^®^/olanzapine rats have similar weights and plasma acetate to controls. The combination of prebiotic and antipsychotic also normalizes gene expression in peripheral tissues.

The parallel changes in hepatic ACC mRNA expression and plasma acetate (Table 2) confirms that the metabolic pathways involving this SCFA are influenced by both prebiotic and olanzapine. This is further corroborated by observing contrasting changes in WAT GPR43 mRNA and plasma acetate, which is intuitive since a homeostatic response to elevations of a substrate could conceivably involve a ‘downregulation’ of its binding receptor. Importantly, previous work has shown that weight gain following a high-fat diet is associated with reduced WAT GPR43 expression, and that the administration of acetate elevated this receptor to control levels^42^. We have observed a similar scenario in the olanzapine administered rats, where those that received the drug alone had a significantly greater body weight and lower WAT GRP43 abundance than the B-GOS^®^/olanzapine group. A detailed investigation of the mechanisms underlying the effects of B-GOS^®^ and/or olanzapine was not the focus of this study, and therefore the role of peripheral SCFA binding receptors, other SCFAs, and genes involved in lipid metabolism and their possible differential responses to prebiotic and antipsychotic, requires further exploration.

It is important to note that a recent study in male mice that used another formulation of GOS administered at a higher concentration and for a longer duration, showed some psychotropic effects but did not reveal changes in acetate levels in the caecum^60^. This suggests that acetate may not be a key mediator of prebiotic effects, and that the changes we observed in the current study (Table 2) were incidental and not related to any causal mechanisms. However, Burokas and colleagues^60^ also demonstrated a reduction of *Bifidibacteria* Spp. and *Lactobacilli* Spp. with GOS whereas we have reported the increase in the former with B-GOS^®^^43,48^(and see Fig. 3). These discrepancies indicate that even the primary actions of these prebiotics are different, and emphasises that our findings with B-GOS^®^ are only applicable to this specific prebiotic mixture, and cannot be extended to all formulations of GOS.

The current analysis of plasma cytokines revealed an elevation of TNFα in B-GOS^®^/olanzapine rats. This may contribute to the reduction in olanzapine-induced weight gain as pro-inflammatory TNFα is a recognised appetite suppressant^32^, and has a significant influence on lipid metabolism in adipose tissue^31^. Although its levels are mildly increased in obesity^61^, studies in animal models of this condition show that the activation of the TNFα system inhibits ongoing weight gain^62^. In view of this evidence, we speculate that the co-administration of B-GOS^®^ and olanzapine led to the release of TNFα, which contributed to the attenuation of weight gain. Given that IL-1β and IL-8 levels remained unaltered in all groups, it is also likely that the change in TNFα was not reflecting an inflammatory state. Clearly, further studies are required to corroborate our observations.

Finally, studies in germ-free mice have shown that olanzapine-induced weight gain is contingent on the presence of gut microbiota^63^, and in female rats, the repeated administration of the antipsychotic increased the relative abundance of the phylum Firmicutes, but reduced Actinobacteria, Proteobacteria, and Bacteroidetes^19^. The latter observations may have been the result of the specific antibiotic actions of olanzapine. The in vitro growth of the Proteobacteria *Escherichia coli* NC101, is completely inhibited by physiological concentrations of olanzapine, whereas the proliferation of the Firmicutes strain, *Enteroccoccus faecalis* OGIRF, is only delayed by the drug but not completely repressed^63^. We have examined specific bacterial genera that are altered by other galacto-oligosaccharides^46^, and have demonstrated that although B-GOS^®^ alone increased *Bifidobacteria *spp., and reduced species within the Firmicutes (*coprococcus*, *oscillibacter*, c*. coccoides, roseburia intestinalis cluster, clostridium XVIII cluster*) and Proteobacteria (*Escherichia/Shigella *spp.) phyla, no effects of olanzapine were observed. The latter discrepancy with other studies might reflect differential duration and dose of olanzapine administration, and/or the method of bacterial analysis (metagenomics vs qPCR in the present study). Nevertheless, it is also worth highlighting that olanzapine attenuated the effects of B-GOS^®^, which suggests that the antipsychotic did influence the gut microbiota albeit when their pre-existing levels were below those of control animals. Additional studies are required to test if the bacteria affected by B-GOS^®^ would proliferate beyond control levels with a longer duration of olanzapine administration at a clinically relevant dose. Our data do not support the involvement of at least some bacterial species in short-term olanzapine-induced weight gain.

In conclusion, the current study demonstrates a strategy to reduce olanzapine-mediated weight gain in schizophrenia without compromising a key central pharmacological action of this antipsychotic. We also demonstrate a NMDAR augmenting effect, supporting a potential pro-cognitive action of the prebiotic. Possible mechanisms of metabolic effects of prebiotics through effects in acetate physiology warrants further investigation.
